# Sterilization of Dental Instruments*Bulletin No. 101 of the Hygienic Laboratory, United States Public Health Service.

**Published:** 1916-06

**Authors:** H. E. Hasseltine

**Affiliations:** Passed Assistant Surgeon, United States Public Health Service


### THE STERILIZATION OF DENTAL INSTRUMENTS.*

H. E. HASSELTINE, PASSED ASSISTANT SURGEON, UNITED STATES PUBLIC HEALTH SERVICE.

The possibility of the transmission of disease from one person to another through the medium of instruments and appliances used in dental operations has long been recognized. Reports of such occurrences are frequently heard from the laity, but authentic reports in scientific publications are practically never published. Few men will deny that such cases do occur occasionally; their frequency, however, is not determinable.

Anyone familar with asepsis has probably noticed errors in the surgical technique of a dentist while receiving dental treatment. Among these errors may be mentioned the placing of sterilized instruments on a swinging tray or glass plate which has not been sterilized, the cleaning of burs on a scratch wheel which has not been sterilized since the burs used on the previous patient have been cleaned thereon, and the frequent handling of the cable of the dental engine, which receives contamination from one patient through the operator's hands and in turn returns a portion of this contamination to the operator's hands when used on subsequent patients. These are some of the errors of technique seen personally, and are mentioned here to emphasize the necessity of having everything which comes in contact with the instruments or hands of the operator free from organisms obtained from previous patients in order to prevent transmission of infection from one patient to another.

A sterile towel over the tray, a scratch wheel which

*Bulletin No. 101 of the Hygienic Laboratory, United States Public Health Service.

can be removed and sterilized with the instruments, and a simple sterile linen covering to put over the cord of the engine would make the operation many times more acceptable to the critic, provided, of course, a fresh towel and covering were used for each patient and the scratch wheel sterilized when each patient vacates the chair.

For improvement of the technique of the future dentists we should look to the dental schools. They all have courses in bacteriology, which, however, in many schools are largely theoretical instead of practical. The morphology and cultural characteristics of various organisms are considered, but their resistance to disinfectants is passed over by noting what certain books say regarding this phase of the subject. Little actual laboratory work is done to show the relative value of the sterilizing agents applicable in dentistry and how their efficiency is tested. As a result the student forms the opinion that any sterilizing agent is effective, and does not learn to check his sterilization by bacteriological tests. For the coming dentists the improvement should come through the schools. For those already in practice it must come through reading and exchange of ideas among the men of the dental and medical professions.

Realizing the necessity of improvement, some members of the dental profession are striving to bring about better sanitary conditions in the offices of dentists at large; also among their patients, by teaching them oral hygiene. This work is most commendable, and to this end the officers of some dental societies have asked the Surgeon-General of the Public Health Service to work out a detailed method for sterilizing dental instruments and appliances, keeping in mind the important factors, simplicity, efficiency, and duration of the process of sterilization. As a result of this request the writer was designated by the Director of the Hygienic Laboratory to consider the matter, make experiments, collect data, and make a report thereon.

The progress of the work has been interrupted frequently by exigencies of the service and the report much delayed as a result.

### The Sterilization of Dental Instruments.

In considering this subject the writer has endeavored to keep within practical bounds rather than to pose as an idealist.

In testing the efficiency of various methods of sterilization of dental instruments, the consideration of spore-forming organisms has been disregarded. A high degree of heat for a prolonged period is required to kill the spores of spore-bearing organisms. Fortunately, the number of such organisms is comparatively few. The vegetating forms of these organisms are killed as easily as are the non-spore bearers. The spore represents a defensive element of the organism against unfavorable influences; that is, though capable of becoming alive, it is apparently in a lifeless phase. Since it remains in the spore stage while in conditions unfavorable for its growth, the spore is easily removed from place to place by mechanical means, thereby increasing the chance of finding a more suitable environment.

By reason of this fact mechanical means of removal of spores from instruments is relied upon. Mechanical cleansing of instruments by a brush or sponge, preferably in running water, will reduce the number of spores so that the number remaining will be too small to consider. For experimental data on the efficiency of mechanical cleansing in the removal of spores, the reader is referred to Francis (1).

Such organisms as the pus-producing cocci, the bacillus of typhoid fever, the bacillus of diphtheria, the bacillus of tuberculosis, the *Treponema pallidum* (syphilis), the bacillus of influenza, the amœbæ, and the thrush fungus, are known organisms that should be considered. To this list may be added the virus of diseases of unknown or uncertain origin, such as that of measles, scarlet fever, mumps, infantile paralysis, and smallpox, all of which seem to be caused by non-spore-bearing organisms.

Of the non-spore-bearing organisms, the staphylococci are reported to be the most resistant to heat, our most efficient disinfecting agent. On this account various strains of staphylococci were used as the test organisms in the experiments made in connection with this investigation.

| Authority | Influenza | Staphylococcus aureus | Streptococcus | Pneumococcus |
|---|---|---|---|---|
| Sternberg (2) | 60°, 5 minutes | Moist, 58°, 10 minutes. Dry, 100° | 54°, 10 minutes. | 52°, 10 minutes |
| McFarland (3) | | 62°, 10 minutes. 80°, 1½ minutes | | |
| Muir and Ritchie (4) | | 80°, 30 minutes. | | |
| Park and Williams (5) | 60°, 5 minutes | 80°, 5 minutes | 54°, 10 minutes. | |
| Hiss and Zinsser (6) | 60°, few minutes | 58°, 10 minutes. Dry, 90 to 100° | 54°, 10 minutes. | 52°, 10 minutes |
| Jordan (7) | | 80°, 30 minutes. | | |
| Abbott (8) | | 62°, 10 minutes. | 54°, 10 minutes. | 52°, 10 minutes |
| Rosenau (9) (10) | 60°, 10 minutes | | 54°, 10 minutes. | 52°, 10 minutes |

| Authority | Meningococcus | Typhoid | Diptheria | Tubercle bacillus |
|---|---|---|---|---|
| Sternberg (2) | | 56°, 10 minutes. | 58°, 10 minutes. | 70°, 10 minutes |
| McFarland (3) | | 60°, 10 minutes. | 58°, 10 minutes. | 60°, 20 minutes |
| Muir and Ritchie (4) | | 60°, 30 minutes. | 60°, 10 minutes. | 70°, 1 hour |
| Park and Williams (5) | Readily | 60°, 1 minute | 60°, 10 minutes. | 80°, 1 minute |
| Hiss and Zinsser (6) | Extr'm'ly sensitive to heat | 56°, 10 minutes. | 58°, 10 minutes. | 60°, 20 minutes 80°, 5 minutes |
| Jordan (7) | | | 55°, 45 minutes. | 60°, 20 minutes |
| Abbott (8) | | 60°, 10 minutes. | 58°, 10 minutes. | 80°, 5 minutes 65°, 15 minutes |
| Rosenau (9) (10) | | 60°, 20 minutes. | Less than typhoid | 60°, 20 minutes |

Table I. shows the thermal death point of various organisms, according to different authorities on bacteriology. It was found that the stock laboratory cultures of staphylococci were much less resistant than strains of the same organism obtained from human beings. For this purpose smears from cases of suspected diphtheria or from cases of tonsilitis were used, thereby getting organisms similar to those encountered in the mouth.

Mixed cultures were used to simulate the conditions found in nature. In a few cases, *Bacillus subtilis,* a non-pathogenic spore-forming organism was encountered.

The resistance of the staphylococci was also tested, when dried on instruments prior to sterilization and when not dried. The resistance was always greater when the contaminated instruments were dry. Consequently the dried infectious material was used in most of the tests.

The physical processes involved in sterilization have been the subject of much research and discussion. It is not necessary to consider here to any extent the theories. They can be stated briefly as the coagulation and hydrolytic theories. The first strives to show that the cell substance of the organism is coagulated by the disinfecting agent, just as the white of an egg is coagulated by heat when cooked. The second theory claims that the death of the organism is brought about by the introduction of water into the cell substance, the consequent hydrolysis being accelerated by the disinfecting agent.

The process of sterilization has been described by Phelps (11) as an increase of the death rate of organisms. But in determining the result of this process there are three factors to be considered, viz: (*a*) The velocity of the death rate of the organisms; (*b*) the time through which it is allowed to proceed; (*c*) the number of organisms present. The product of these three factors gives the result. Sterilization is, therefore, a relative result from a mathematical viewpoint and is usually arbitrarily recorded as perfect when the number of bacteria in the volume tested is less than one. On this death rate of organisms, the number present, and the time allowed, depend the efficiency of a sterilizing agent.

However, the exact mode of killing the organisms does not interest the practicing dentist and surgeon so much as does the assurance that all will be killed, in some manner, under certain conditions.

The methods of sterilization employed at present in most dental offices may be classified as follows:

| | | | |
|---|---|---|---|
| Thermal | Boiling in water.<br>Use of boiling water.<br>Passing an instrument through a free flame.<br>Moist heat in a closed or open chamber.<br>Dry heat in a closed chamber. | Chemical | Carbolic acid, followed by alcohol.<br>Other coal-tar products, followed by alcohol.<br>Formaldehyde vapor in a tight chamber.<br>Other chemicals, such as gasoline, solution of biniodide of mercury. |

The efficiency of most of these methods was tested, and the advantages and disadvantages of each considered.

### Boiling in Water.

*Results of experiments.*—Most authorities state that non-spore-bearing organisms are killed by boiling for ten minutes. A few have reported organisms of this class that have resisted boiling for thirty to sixty minutes, but such are too infrequent occurrence to require consideration. Of the various strains of staphylococci in mixed culture, which I have tested, I have found none that survived three minutes boiling. This means exposure to water heated until noticeable ebullition takes place before the organisms are placed therein, the time being taken by the watch. Undried instruments were sterilized in two minutes. Sterility tests were made by dropping the sterilized instruments into tubes of nutrient bouillon and incubating these for forty-eight hours at thirty-seven degrees. Though my experiments show sterilization in a shorter time, a period of ten minutes boiling is recommended, to provide an ample margin on the side of safety. A small amount of alkali, such as sodium carbonate, or sodium hydroxide, should be added to the water to prevent rusting of instruments.

The advantages of this method are: (*a*) It is the most rapid and most efficient; (*b*) it is always available where fire and water can be obtained; (*c*) the procedure is simple and can be carried out by any one; (*d*) the expense of sterilization by this method is small.

The disadvantages are: (*a*) Dulling of the sharp edge of cutting instruments, such as knives, lancets, etc.; (*b*) increase of effect of action of any chemical impurity in the water upon the instruments, a factor that will vary according to local conditions.

### Use of Boiling Water.

*Results of experiments.*—If used immediately after the heat is cut off, and in quantity sufficient to guard against a rapid cooling, this method is nearly the equivalent of boiling, so far as practical results are concerned. The use of one gallon of water, boiled until the moment before the instruments are submerged in it, sterilizes as rapidly as does boiling, at least during the first five minutes of exposure. If care be taken to carry out this procedure fully, the method is efficient, the advantages and the disadvantages being the same as with boiling, save that the rapid cooling of the water will necessitate reheating to the boiling point to insure proper results.

The use of water of too low temperature or for too short a period of time makes possible incomplete sterilization. As the temperature is lowered the period of exposure must be lengthened; but, as the loss of heat is uncertain, the required length of exposure will necessarily be uncertain. On account of the uncertainty of the temperature of the water, except it be boiled immediately prior to use on each occasion, this method is not as good as boiling or the use of a *known* temperature at a degree lower than the boiling point.

### Passing an Instrument Through a Free Flame.

This method is effective, since, if properly carried out, it virtually amounts to incineration of the organisms. The degree of heat to which the instrument is subjected is much higher than the boiling point, and on this account only a few instruments can be sterilized by this method without interfering with cutting qualities, temper of steel, or plating of instruments. It is applicable to certain small instruments whose cost is not high, but is not available for general use.

### Moist Heat in an Open or Closed Chamber.

This is applied by the use of steam in an open chamber (Arnold) or in a tight chamber under pressure (autoclave). Its use for instruments is not very extensive, as the same results can be obtained by direct boiling and some time saved, since boiling a small amount of water requires much less time than heating up a large apparatus. It is of greatest value for sterilizing linens, gowns, towels, etc., and the steam under pressure is preferable, on account of its greater penetration.

### Dry Heat in a Closed Chamber.

This method is not used for instruments, on account of the high temperature required and the long time required to carry it out. It can be used for glassware or for sterilizing linen, if the temperature be not too high.

### Use of Carbolic Acid Followed by Alcohol.

This method consists of submersion of the instruments in phenol of varying strength. The solubility of phenol in water is about one part in twenty, giving a five per cent. solution. This solution is more effective than the pure phenol. After submitting the instruments to this treatment they are immersed in alcohol, to remove the excess of phenol, and may then be rinsed in water, or used without rinsing.

*Results of experiments.*—With many strains, ten minutes' exposure to five per cent. phenol, followed by one minute in alcohol, sufficed to sterilize. Some strains of staphylococci, however, were more resistant and uniformly gave growth after fifteen minutes; in a portion of the tubes even after thirty and forty-five minutes'

exposure. No strain used survived one hour in five per cent. phenol. The same resistant strain was uniformly killed by boiling for three minutes. It was also killed by three minutes' exposure in an eighty degree water bath.

Its advantages are: (*a*) The cost is moderate; (*b*) it can be applied to practically every instrument and appliance without damage resulting therefrom.

Among its disadvantages are: (*a*) It requires a longer period of time to sterilize; (*b*) it requires more care in the application of the method (care must be taken to see that all surfaces of instruments are exposed to the solution); (*c*) instruments must be removed from phenol by forceps, as the solution had a bad effect on the skin if the hands are used in it frequently; (*d*) it requires a much better trained assistant, or the attention of the dentist himself, to use this method properly; (*e*) the odor of phenol is, to some, objectionable.

The use of other coal-tar products resembles that of phenol in application and efficiency. The advantages and disadvantages mentioned under phenol apply also to these products, except as follows: (*a*) Some have a higher co-efficient than phenol; that is, they are more germicidal than phenol; (*b*) some have a soapy composition, giving them the cleansing property of soap when mixed with water; (*c*) their cost, however, is in most cases considerably greater.

### Formaldehyde Gas in an Air-tight Chamber.

This method has, of late years, been very popular among dentists and is efficient, provided a sufficient length of time is allowed for action.

*Results of experiments.*—Exposure of contaminated instruments to formaldehyde vapor in an air-tight glass jar for periods varying from ten minutes to one hour gave growth more or less constantly. Some strains of staphylococci were always killed by thirty minutes' exposure; the more resistant strains survived for sixty minutes. An exposure of one and one-half to two hours killed the most resistant strain used. These results were obtained with an excess of formalin in a small glass jar closed by ground-glass joints sealed with vaselin, so the air was filled to saturation with the formaldehyde vapor.

The advantage of this method lies solely in its simplicity and ease of application.

Its disadvantages are: (*a*) The time required to sterilize is too long for a busy dentist; (*b*) formaldehyde causes quite a marked rusting of unplated steel instruments; (*c*) inefficiency is likely to result from the lessening of the density of formaldehyde vapor, unless special care be taken to replenish the supply of formalin solution at frequent intervals; (*d*) inability to remove a single instrument from the air-tight chamber without interfering decidedly with the process of sterilization, because of the lessening of the density of the formaldehyde vapor in the chamber; (*e*) the disagreeable odor and irritating effect on mucous membranes will be experienced more or less by both operator and patient.

### Other Chemical Methods.

Disks containing mercuric iodide with some alkaline salt are theoretically practicable. The alkalinity is supposed to guard against the corrosive action of the mercury on the metal instruments. However, this brings a rather delicately balanced chemical reaction into use, and unless the application of the method be carried out by the dentist himself, or an assistant who has some knowledge of chemistry, bad results may be obtained. For instance, the chemical impurities of the water used may vary from day to day, and thus disturb the proper degree of alkalinity. No experiments were made with these disks, as it is not conceivable that their use is an improvement on some of the simpler and less technical methods.

The action of various chemical substances, such as gasoline, petroleum-ether, etc., was tried by the writer. Their efficiency is doubtful and uncertain. No sterilization was obtained except on prolonged exposure (twenty-four hours or more).

In addition to these methods the writer tried the application of moist heat at a constant degree, lower than the boiling point; eighty degrees was the temperature used. This was tried in order to get a method that would interfere less with the edge of cutting instruments than does boiling; also, a method that could be applied to certain instruments that could not be boiled without marked deleterious effect on them, such as dental mirrors.

A jacketed water bath, designed by Mr. W. F. Wells, which was equipped with an apparatus for maintaining a constant level of water in the jacket, was used for this purpose. The inner chamber contains a thermoregulator set at eighty degrees. In this bath the contaminated instruments were placed for varying periods.

To alkalinize the water, 0.25 per cent. sodium hydroxide was added. When once raised to eighty degrees, the amount of gas or electric current required to maintain a bath containing one to two gallons is very small, indeed. In fact, it can be maintained at that temperature for the whole twenty-four hours cheaper than it can be extinguished and reheated each day.

The use of this degree of heat had no deleterious effect on metal instruments or dental mirrors after prolonged and repeated exposure. Sterilization was always obtained in three minutes, even with the most resistant strains of staphylococci used. It is thus seen that this is practically as effective as boiling, but the temperature is twenty degrees lower. It is simple to operate when once installed and is very efficient and rapid.

As stated above, no bad effects were noted on dental mirrors or instruments. However, there was a bad effect on instruments with wooden or hard-rubber handles. In the present era, however, no instrument that is not entirely of metal has any place in the armamentarium of surgeons or dentists. There are a few exceptions to this rule, where flexibility or elasticity is a factor in the use of the instrument.

From the above experiments and considerations the methods depending upon moist heat were found to be by far the most rapid and efficient.

In order of merit the writer places the methods depending upon heat as the active disinfecting agent as follows:

1. Boiling for at least ten minutes in 0.25 per cent. sodium hydroxide.
2. Use of water bath at eighty degrees for at least ten minutes.
3. Use of moist heat in free chamber (Arnold sterilizer) for at least ten minutes after thermometer reaches 100 degrees.
4. Submersion in boiling water for at least ten minutes, the source of heat being removed immediately prior to submersion of the instrument.
5. Application of dry heat by passing instrument through a free flame.
6. Dry heat in closed chamber.

The arrangement of the chemical methods in order of merit is much more difficult; in none are simplicity, efficiency, and rapidity combined as in some of the methods dependent on heat.

Using formaldehyde, the simplicity is ideal; but the length of exposure necessary to gain efficiency makes it undesirable. With phenol and other coal-tar products efficiency is good but simplicity and rapidity are partially sacrificed. With mercuric iodide, the same factors that militate against phenol, plus a possible deleterious action on the instruments, are applicable.

It is the opinion of the writer, therefore, that in selecting a method for adoption in dental offices the use of moist heat is essential. Some will offer the objection that a dentist has many instruments which will not stand such procedure. I will admit that there are, perhaps, a few instruments which can not be boiled without an injurious effect on them; but after eliminating hard rubber and wood handled instruments, which have no place in any instrument case of the present age, this list becomes very small.

I shall now consider the method of choice as applied to different instruments.

The instruments in the following list can all be boiled without marked bad effects unless the water used has some special chemical impurity which attacks the metal. Such conditions will have to be determined for each water supply and the proper remedy sought through scientific channels.

Broaches and their holders.
Burnishers.
Burrs.
Chisels.
Cone-socket instruments with metal handles.
Drills.
Excavators.
Explorers.
Files.
Forceps, extracting.
Forceps, foil.
Forceps, tongue-holding.
Impression trays.
Knives and lancets.
Mallets, automatic and hand.
Mixing slabs.
Mouth gags.
Mouthpiece of gas apparatus.
Mouthpiece of saliva ejector.
Pluggers.
Polishing points.
Pyorrhea instruments.
Root elevators.
Rubber-dam clamps, and forceps for the same.
Rubber-dam holder (metal parts) and weights.
Saws.
Scissors.
Scalers.
Spatulas, metal.
Syrines, water.
Syringes, hypodermic.

The latter should be of glass or metal without washers; they can then be boiled repeatedly without impairing their efficiency in any way. An inferior hypodermic syringe is always unsatisfactory, whether it be sterilized or not.

To the above list I will add the following as instruments that will stand boiling.

Chip blower.

Masks for giving nitrous oxide gas.

Wire scratch wheel for head of dental engine.

Engine hand pieces.

The wire scratch wheel on the head of the dental engine should be made entirely of metal and detachable, so that it can be easily removed and boiled. Probably a cheap bristle wheel might be made that could be discarded after each patient is treated.

Probably many will challenge the statement that engine hand pieces can be boiled. This statement is made only after having procured a right-angled hand piece, boiled the same, and tested its sterility, then contaminated it with a fluid culture of bacteria, and again boiled it and tested its sterility. After completing the experiments with it for the day it was placed in ninety-five per cent. alcohol to remove the excess of water, and in several instances left in this fluid over night. It has been boiled repeatedly and frequently exposed to eighty degrees in a water bath, yet it has shown no bad effects as a result of such treatment, except the removal of the lubricant from the mechanism. It can be oiled in a few seconds and its mechanism will run as smoothly as before. These hand pieces could be made with oil holes at the proper points so that the oiling would be much facilitated.

This instrument is one which dentists have usually considered nonsterilizable, consequently it has received very little efficient sterilization. Marshall (12) states that hand pieces can be sterilized by immersing them in gasoline and then in alcohol; but my experiments with sterilization by anhydrous liquids have given disappointing results, even with instruments of simple construction. Furthermore, the effect of gasoline and alcohol on the lubricant will be practically as complete a removal of the oil as when boiled, so that oiling of the piece would be necessary after each sterilization.

In sterilizing hand pieces, heat will reach infection in parts of the instrument where cold solutions can not be effective, on account of the oil present, and where gas does not penetrate. With heat, the metal itself is raised to the temperature of the surrounding medium, so that the organisms are attacked from all sides; while with solutions or gases more or less of their surface is protected.

In seeking a method of sterilization for hand pieces, several methods were tried, designed to obtain sterilization without the introduction of water into the instrument. As stated above, the gasoline method did not sterilize rapidly and completely. Immersing in a fixed oil, liquid vaseline was then tried, placing this in a steam chamber and raising the temperature to 100 degrees for one hour. This proved inefficient, because the heat is only the equivalent of 100 degrees of dry heat, the instrument being in an anhydrous fluid. As a last resort I turned to moist heat by the method of boiling, or an eighty degree water bath, in 0.25 per cent. sodium hydroxide and then removed the excess of water by absorbing it with alcohol. This gave the desired result.

All of the above mentioned instruments may be submitted to eighty degrees in a water bath without any injurious effect on the instruments. Mouth mirrors may also be sterilized by this method without injuring them. This was determined by obtaining several new mirrors, contaminating them, and then placing them in 0.25 per cent. sodium hydroxide solution at eighty degrees. Sterilization was accomplished in three minutes, though the mirrors were frequently left in the bath for a much longer period, ten to sixty minutes. The test of the effects of this treatment was made by handing the mirrors, together with a new mirror of the same lot that had not been sterilized, to other persons and asking them to pick out the one that had not been sterilized. Everyone acknowledged his inability to determine any difference.

Boiling, or heating to eighty degrees, the rubber bulbs of chip blowers, and the face piece of the gas inhaler, will shorten the life of these articles. It is believed, however, that the increased expense, due to these rubber fittings, will not be great enough to work any hardship on the dentist. It certainly will not be as great as the cost of rubber gloves used by the average surgeon; and at present nearly every surgeon uses rubber gloves and insists on their sterilization by boiling. The dental surgeon should not be content with a lower grade of work, nor deterred by the small sum necessary to replace articles which have been worn out a little sooner by reason of improved methods in his practice.

The instruments considered above, which can be sterilized by boiling, include the greater portion of those used by the dentist; except those whose cost is so slight, or whose usefulness so impaired after using once, that they can be thrown away.

This list includes:

Polishing points and disks.
Rubber dam.
Nerve broaches (these may be sterilized by heat if desired).
Bristle brushes.

Mounted stones must be treated according to their construction and composition. Carborundum disks, wheels, and stones, which are bonded with porcelain, can be boiled without injury. Those made with rubber as the adhesive base can not be heated. Their sterilization can be accomplished by use of a phenol solution followed by alcohol. Corundum stones can not be boiled, so the phenol solution should be used on these. Stones mounted on a mandrel by a cement or shellac can not be subjected to heat, while those mounted by metal devices can be boiled, provided their composition allows such treatment. Inasmuch as many of the stones and disks must be sterilized without heat, it is permissible to sterilize all stones by immersing in five per cent. phenol solution for one hour, to be followed by immersion in alcohol to remove the excess of phenol solution.

Tortoise shell and instruments of similar composition, which might be injured by heat, should be immersed in phenol solution, as are mounted stones. Mouth mirrors may also be sterilized in this manner, if not subjected to eighty degrees.

The life of a mouth mirror, however, depends entirely on the efficiency of its construction in keeping fluids of any kind from the amalgam surface of the glass. As long as this can be kept dry the mirror is clear. When it becomes wet by any fluid, either water or saliva, it becomes cloudy, consequently a well-constructed mirror is the cheapest in the end.

This method may also be applied to instruments whose composition must be other than metal, on account of the discoloring effect of metal on cement, or for other good reason.

However, barring the few instruments whose chemical composition is such that a serious change in character would result from heating, all instruments should be sterilized by heat, and no instrument made of material that is destroyed by heat should be allowed in the instrument case, if the same instrument can be made of thermostable matter.

I have seen some dental instruments with rubber handles which have been boiled repeatedly, with no bad effect except discoloration of the rubber. These, however, had a central metal shaft supporting the rubber, to prevent bending when heated. While this construction permits the boiling of rubber-handled instruments, it can not be said that rubber handles are as acceptable from an aseptic viewpoint as are all-metal instruments. Of course, instruments whose cost is so slight that they are used once and then discarded, do not require sterilization to prevent transfer of infection from patient to patient.

### Other Sanitary Measures.

The following suggestions relating to dental asepsis, while not strictly within the subject of sterilization of instruments, are given as contributory factors in improved office sanitation.

Cuspidors are a necessity in a dental office, and the problem of keeping them in sanitary condition is a difficult one. The installation of the fountain cuspidor has been a great improvement over the old metal or earthen bowls which were so hard to keep clean. To those dentists who still have the old type of cuspidor, on account of lack of running water or for other reason, I would recommend at least daily cleaning, with a broom or stiff brush and water. This is to be followed by placing the cuspidor in a bucket, covering it with boiling water, and leaving it there for at least one hour. It would be well to have several of these, so that a clean one can be put in use for each patient while the others are being cleansed by assistants or char-women. In offices having the single-bowl fountain cuspidor, with bowl that can not be removed, flushing with hot water and antiseptic solutions is our main effort in sterilization. All fountain cuspidors should be fitted with both hot and cold water, so that the hot water can be used for cleaning the bowls after each patient is dismissed. Those having bowls that can be removed can have the bowls immersed in antiseptic solutions, or may even be boiled in a large container. However, absolute sterility of cuspidors is not of vital importance, provided a high degree of cleanliness is maintained. It is well, however, to have these articles of equipment installed so that complete sterilization can be carried out in case an unusual infection, such as diphtheria, is encountered.

The possibility of the operator transferring infection to the cable of the dental engine above the hand piece, and then later from the cable to another patient, has been mentioned. If the cables have the power-transmitting mechanism inside a covering, this can be avoided by a simple sterile or clean cotton sleeve, with a draw string in each end, which can be slipped over the cable and tied after the hand piece is in place, so that the whole cable is covered down to the sterile hand piece. This allows the operator to handle the cable at will during his operation, and, when a second patient is treated, a second clean sleeve is put on in place of the first one. It is also probable that a flexible metal covering, of tubing such as is used for conducting gas to lamps, might be constructed so that this could be removed and sterilized by heat. This is not applicable to engines which transmit the power from engine head to hand piece by open cord belt.

The use of foot levers for turning on the flow of hot and cold water in the washbowl is a great convenience to a busy operator who desires to keep his hands sterile. The use of a clean washable covering for the operating chair is a step in the direction of asepsis and cleanliness. The headrest should have a clean linen cover, renewed for each patient.

There should be toilet facilities for patients and, in addition to the usual lavatory equipment, a dental bowl, like that seen on Pullman cars, so that a patient may brush his teeth without doing so over the washbowl.

To place the patient's mouth in as clean condition as possible, each patient should brush his teeth, preferably immediately prior to coming to the chair. This should be followed by a thorough rinsing of the mouth with some good mouth wash. This procedure is a mechanical cleansing, aimed primarily at the removal of spores from the field of operation, though many other organisms are removed at the same time.

In order to do this the dentist should have a supply of toothbrushes on hand and, if necessary supply a new brush to each patient. This may be the patient's first encounter with the oral hygiene educational movement, but I believe it will be productive of good. I believe that the dentist should add the cost of the brush to the patient's bill, unless brushes can be obtained at so low a figure as to make their free distribution practicable. A supply of mouth wash should be on hand in a convenient place for use by patients.

Paper drinking cups and paper, or small individual, towels for patients should be furnished. The common drinking cup and towel are abolished on all interstate carriers, and in hotels and public places in many States. It is, therefore, fitting that the members of the medical and dental profession should apply the spirit of these laws to their own establishments, even though the letter of the law does not require it.

Clean linen direct from the laundry is not sterile, but offers only the remotest chance for transmission of infection through it. Sterile linen is desirable and for a busy dentist, apparatus for its sterilization will be a great addition to office equipment. Steam pressure sterilizers are preferable for this, though other types may be used.

### Summary.

As a result of the experiments and investigations detailed above, the writer reaches the following conclusions and recommends the following methods for sterilization of dental instruments:

### Conclusions.

1. Moist heat is our best disinfecting agent for the sterilization of all metal instruments.

2. For the destruction of non-spore-bearing bacteria, moist heat at eigthy degrees is nearly as efficient as boiling, and for practical purposes can be used in place of boiling.

3. Instruments constructed of metal, whose complicated mechanism has heretofore caused them to be considered as non-sterilizable, can be sterilized by moist heat, provided the water is removed from them by immersing in alcohol subsequent to sterilization.

4. Instruments, whose construction does not permit of boiling, can be sterilized by chemical disinfectants.

5. There is need for more practical instruction in dental schools and clinics in the methods of sterilization, and the subsequent testing of the same by bacteriological methods.

6. Dentistry, which is a highly specialized branch of surgery, should use the two factors, asepsis and anesthesia, which have made possible the wonders of modern surgery, with skill and precision equal to that of surgeons.

### RECOMMENDATIONS.

1. That all instruments and appliances be rendered mechanically clean by washing in water with a brush or sponge.

2. That the following instruments and appliances be boiled or submitted to eighty degrees in a slightly alkaline solution (0.25 per cent. sodium hydroxide):

Artificial teeth used in matching and measuring.
Broaches and their holders.
Burnishers.
Burrs.
Chip blowers.
Chisels.
Drills.
Excavators.
Explorers.
Files.
Forceps, extracting.
Forceps, foil.
Hand pieces for engine.
Impression trays.
Knives and lancets.
Mallets, hand and automatic.
Mixing slabs.
Mouth gags.
Mouthpiece of saliva ejector.
Pliers.
Pluggers.
Pyorrhea instruments.
Polishing points and brushes (if not discarded after using once).
Reamers.
Root elevators.
Rubber dam clamps and forceps for same.
Rubber dam weights and metal parts of holder.
Saws.
Scalers.
Scissors.
Scratch wheel on head of engine.
Spatulas, metal.
Syringes, hypodermic.
Syringes, water.
Tongue-holding forceps.
Mirrors (if eighty degree bath be used, but not to be boiled).

3. That instruments in the above list whose mechanical construction makes it difficult to remove the excess of water are to be placed in ninety-five per cent. alcohol for ten minutes to remove water, then removed and allowed to dry.

4. That only instruments with metal handles be used by dentists desiring to follow this method.

5. That the following instruments be sterilized by immersion in five per cent. solution of phenol for at least sixty minutes:

Mounted stones.
Tortoise-shell instruments.
Mirrors (when eighty degree bath is not used).
Other instruments not of metallic nature and which can not be replaced by metallic instruments.

6. That instruments, after using, be placed in a fluid medium, preferably clean water, to avoid drying of infectious material and to facilitate their mechanical cleansing.

7. That no instrument or appliance, used on a patient directly or indirectly, be used on any other patient until recommendations 1 and 2, or 1 and 5, as the case may be, have been complied with.

### Bibliography.

1. Francis, E. Laboratory studies upon Tetanus. (Hyg. Lab. Bull. No. 91, p. 70.)
2. Sternberg, George M. Text book of Bacteriology, 1901.
3. McFarland, Joseph. Text book upon the Pathogenic Bacteria, 1909.
4. Muir and Ritchie. Manual of Bacteriology, 1907.
5. Park and Williams. Pathogenic Microorganisms, 1914.
6. Hiss and Zinsser. Text book of Bacteriology, 1914.
7. Jordan, Edwin O. General Bacteriology, 1909.
8. Abbott, A. C. Principles of Bacteriology, 1901.
9. Rosenau, M. J. Disinfection and Disinfectants, 1902.
10. ———. Hygiene and Preventive Medicine, 1912.
11. Phelps, Earle B. J. Infect. Dis., Chicago, 1911, 8: 27.
12. Marshall, J. M. Items of Interest, New York, 1912, 35:765.

